# In-hospital clinical complications of COVID-19: a brief overview

**DOI:** 10.2217/fvl-2021-0200

**Published:** 2021-11-04

**Authors:** Kevin John John, Jemimah Nayar, Ajay Kumar Mishra, Vijairam Selvaraj, Mohammad Saud Khan, Amos Lal

**Affiliations:** ^1^Department of Critical Care, Believers Church Medical College Hospital, Thiruvalla, Kerala, India; ^2^Department of Nuclear Medicine, Christian Medical College, Vellore, India; ^3^Department of Cardiovascular Medicine, Saint Vincent Hospital, Worcester, MA, USA; ^4^Department of Medicine, The Miriam Hospital & Warren Alpert Medical School of Brown University, Providence, RI 02906, USA; ^5^Department of Cardiology, University of Kentucky at Bowling Green, Bowling Green, KY 42102, USA; ^6^Department of Medicine, Division of Pulmonary, Critical Care Medicine and Sleep Medicine Mayo Clinic, 200 First Street Southwest Rochester, Rochester, MN 55905, USA

**Keywords:** complications, COVID-19, hospital, mortality, multicenter, outcomes, virus

COVID-19 caused by SARS-CoV-2 originated from Wuhan, China and led to a pandemic that has resulted in more than 4.13 million deaths worldwide. While this is a staggering statistic, the number of deaths alone does not fully capture the impact of this disease. Numerous short- and long-term complications involving almost all organ systems have been reported in COVID-19 patients. Data from a prospective, multi-center cohort study in 302 UK healthcare facilities suggests that 49.7% of patients admitted with COVID-19 have at least one in-hospital complication [1]. The most commonly involved systems were renal (24.3%), respiratory (18.4%), cardiovascular (12.3%), neurological (4.3%) and gastrointestinal (0.8%). Therefore, it is essential to recognize that COVID-19 is not just a respiratory illness, rather a multi-system disease that requires multi-disciplinary care. The long-term sequela of these complications is beyond the scope of this manuscript and will be discussed in the subsequent paper(s).

## Complications in critically ill COVID-19 patients

One of the most common in-hospital complications seen in critically ill COVID-19 patients is barotrauma which can be in the form of a pneumothorax, pneumomediastinum or subcutaneous emphysema [2]. Barotrauma has an incidence that ranges from 7.4 to 40% in patients requiring invasive mechanical ventilation (IMV), and between 4.7 and 8.1% for noninvasive ventilation [3]. An observational study of 116 invasively ventilated patients noted a relationship between barotrauma and worse lung involvement at intensive care unit (ICU) admission. The same study also observed higher mortality (60.7% vs 38.6%; p = 0.04) in patients with these complications [4]. Lemmers *et al.* observed more cases of pneumomediastinum or subcutaneous emphysema in COVID-19 patients with acute respiratory distress syndrome (ARDS) than those without (13.6 vs 1.9%; p < 0.01) and argue that lung frailty, not barotrauma is the cause of these complications. This is because these complications seem to occur despite the use of lung-protective ventilatory strategies such as low tidal volume and plateau pressure [5]. There are several postulated mechanisms for this complication. Cytokine-mediated alveolar injury may lead to the release of air (the ‘Macklin effect’), which then tracts along the broncho-vascular tree toward the hilum leading to pneumothorax or pneumomediastinum [3]. Second, significant inflammation may lead to pneumatocele formation, rupture and also subpleural necrosis leading to the occurrence of bronchopleural fistulas. Third, increased alveolar pressure and diffuse alveolar injury along with low compliance also make the alveoli more prone to rupture. Last, dexamethasone may also play a role in spontaneous pneumothorax by delaying healing and perpetuating air leakage. It is therefore not surprising that several cases of spontaneous pneumothorax, pneumomediastinum and subcutaneous emphysema have been reported in ambulatory COVID-19 patients [6,7]. Hence the consideration of these complications should not be restricted to hospitalized patients alone. Despite the initial surge of misinformation, it is of crucial importance to comply with the best clinical practices that have been established for the care of these critically ill patients based on sound clinical research from the past.

Critically ill patients are also at increased risk for sepsis. A rapid review estimated that 8% of COVID-19 patients have bacterial co-infection, while 72% of hospitalized COVID-19 patients received at least one antimicrobial agent [8]. According to a review by Chong *et al.*, the incidence of secondary pulmonary bacterial and fungal infections in hospitalized COVID-19 patients was 16% (4.8–42.8%) and 6.3% (0.9–33.3%) respectively, and were most common in critically ill patients [9]. While these numbers vary greatly depending on the geographical location, they may indicate antibiotic use without a strong indication. The widespread use of broad-spectrum antibiotics may also accelerate the emergence of multidrug-resistant strains. Some of the risk factors for bacterial co-infection are intestinal colonization by carbapenem-resistant enterobacteria (OR: 16.03; 95% CI: 6.5–39.5%), invasive mechanical ventilation (OR: 5.6; 95% CI: 2.4–13.1%), immunomodulatory agents (OR: 5.09; 95% CI: 2.2–11.8), C-reactive protein on admission >7 mg/dl (OR: 3.59; 95% CI: 1.7–7.7%) and previous treatment with piperacillin/tazobactam (OR: 2.85; 95% CI: 1.1–7.2%) [10].

The pooled proportion of fungal infection in COVID-19 patients is estimated to be 0.12 (95% CI: 0.07–0.16) and the pooled proportion of mortality in this group is estimated to be 0.17 (95% CI: 0.10–0.24) [11]. The risk of fungal infection is compounded by the increased use of immunosuppressive medications such as steroids, and this was demonstrated by the recent epidemic of COVID-19-associated rhino-orbital-cerebral mucormycosis in certain developing countries [12]. COVID-19-associated pulmonary aspergillosis (CAPA) is also of concern and has an incidence of 13.5% (2.5–35.0%) among hospitalized patients [13]. The overall mortality associated with this condition is also high (48.4%). Given these observations, the authors are of the opinion that strict antibiotic stewardship must be emphasized at all centers managing COVID-19 patients. Appropriate duration and dosing of steroids continues to be a matter of debate and further research. The use of IL-6 inhibitors and other immunomodulating agents have proven to be useful in this subset of COVID-19 patients. Related adverse events (AEs) have to be closely monitored and should be reported appropriately.

## Cardiac complications

According to a meta-analysis, the most common cardiac complications in COVID-19 were myocardial injury (21.2%; 95% CI: 12.3–30.0%), arrhythmia (15.3%; 95% CI: 8.4–22.3%), heart failure (14.4%; 95% CI: 5.7–23.1%) and acute coronary syndrome (1.0%; 95% CI: 0.5–1.5%) [14]. Patients with cardiac injury have higher mortality than those without (71.2 vs 6.6%; p < 0.001) [15]. The prevalence of myocardial infarction with nonobstructive coronaries (MINOCA) seems to be higher in COVID-19 patients, as observed by Bangalore *et al.* [16]. These complications may be an interplay between traditional risk factors and the systemic inflammation triggered by COVID-19 infection [17]. The role of ACE-2 receptor and the cardiotropic properties of SARS-CoV-2 cannot be ignored either [18,19].

Zylla *et al.* reported that 3% of patients developed newly diagnosed (*de novo*) left ventricular dysfunction after admission, while Zhou *et al.* and Chen *et al.* reported a number close to 25% [20–22]. This is concerning because heart failure can worsen acute respiratory distress syndrome because of heart–lung interactions and pulmonary mechanics affected by the fluid shifts. Myocarditis and Takotsubo cardiomyopathy are also seen in COVID-19 patients and it can be difficult to differentiate between the two [17]. However, this differentiation has therapeutic and prognostic implications. For example, in patients with Takotsubo syndrome, it may be necessary to avoid catecholaminergic vasopressors and inotropic agents. Right ventricular (RV) dysfunction has been identified in COVID-19 infection with a rate of 30% noted in several reports. RV dysfunction may arise from left ventricular dysfunction in the setting of myocarditis or acute coronary syndrome. The RV may be subject to direct myotoxic effects of the virus and thrombotic microangiopathy accompanied by micro- and macro-thrombosis in the pulmonary vasculature leading to RV volume overload [23].

Arrhythmias are also common in hospitalized COVID-19 patients due to a variety of reasons. According to one study, the most commonly reported tachyarrhythmia was atrial fibrillation followed by atrial flutter, while severe sinus bradycardia was the most common bradyarrhythmia [24]. The use of QT-prolonging drugs such as macrolides and hydroxychloroquine can further accentuate the risk of arrhythmia. It is crucial to address these risk factors because COVID-19 patients with arrhythmia have an increased risk of severe disease, ICU admission and poor outcome [25]. Therefore, the authors recommend a daily review of medications to reduce drug interactions and other complications due to polypharmacy.

Hospitalized COVID-19 patients are also at risk of complications such as pericardial effusion, tamponade and left ventricular thrombosis [26–28]. However, the frequency of occurrence of these complications is much lower than the ones mentioned prior.

## Neurological complications

There is growing evidence to support the hypothesis that SARS-CoV-2 is a neurotropic virus [29,30]. The virus may reach the nervous system through retrograde axonal transport or hematological dissemination. Moreover, the procoagulant state seen in COVID-19 may result in thrombotic and embolic complications involving the nervous system.

The average incidence of stroke in a meta-analysis of 55,176 COVID-19 patients was 1.74% (95% CI: 1.09–2.51%) and the average mortality of stroke in COVID-19 was 31.76% (95% CI: 17.77–47.31%) [31]. Case reports and case series of cerebral venous thrombosis (CVT) have also been described in the literature [32]. However, the most common neurological complication in COVID-19 patients is probably delirium which is seen in as many as 55% of hospitalized patients [33]. Rarer central nervous system complications that have been described are encephalitis, myelitis, meningoencephalitis, reversible cerebral vasoconstriction syndrome and acute disseminated encephalomyelitis (ADEM) [30,34,35]. Reports of Guillain–Barré syndrome (GBS) and GBS variants after COVID-19 are emerging [36]. Psychiatric complications such as depression, impulsivity, acute stress disorder, insomnia and post-traumatic stress disorder are also common in hospitalized COVID-19 patients, especially those admitted to the ICU [37]. Such insults must be identified with appropriate screening tools and addressed during the rehabilitation of COVID-19 patients [38].

## Hematological complications

Changes in hematological indices can be seen in 20–50% of hospitalized COVID-19 patients, who may develop both thrombotic and hemorrhagic complications [39]. Increased inflammatory response, hypoxia, immobilization and disseminated intravascular coagulation (DIC) can lead to both venous and arterial thromboembolism. An Italian study of 388 patients found such events in 27.6% of patients in the ICU and 6.6% of patients in the general wards, despite the use of thromboprophylaxis in all ICU patients and three-quarters of ward patients [40]. ICU hospitalization, higher leukocyte count, higher neutrophil/lymphocyte ratio and higher d-dimer value are some of the risk factors for the development of venous thromboembolism [41]. Therefore, thromboprophylaxis should be considered in all hospitalized COVID-19 patients in the absence of risk factors for bleeding. We would urge clinicians to use clinical risk scores such as the ‘Padua score’ to guide decision-making in this regard. However, these decisions may be complicated by thrombocytopenia, which is seen in around 36.2% of COVID-19 patients [42]. The underlying prothrombotic state and endothelitis may be responsible for acute limb ischemia being five-times as common in COVID-19 patients compared with the general population [43]. Forty percent of those who develop acute limb ischemia die, according to an Italian study [44].

## Renal complications

Acute kidney injury (AKI) has a pooled incidence of 12.3% in hospitalized COVID-19 patients [45]. Moreover, COVID-19-related AKI is associated with poor disease outcomes and higher mortality (72 vs 42%; p < 0.01) [46]. The causes of AKI in COVID-19 can be multifactorial and include direct and indirect effects of viral infection, drugs, sepsis, ischemia, rhabdomyolysis, thrombotic events and other organ dysfunction [47]. What is also noteworthy is that a random-effects meta-analysis of 14 studies with 17,876 patients showed that renin–angiotensin–aldosterone system (RAAS) blockade significantly increased the risk of AKI in hospitalized COVID-19 patients (OR: 1.68; 95% CI: 1.19–2.36); an association that remained significant after stratifying by drug class and AKI severity [48]. The cause of renal injury may be glomerulonephritis, thrombotic microangiopathy, tubular injury or interstitial nephritis [49]. The possibility of drug-induced AKI must also be ruled out.

## Gastrointestinal & hepatobiliary complications

The most devastating gastrointestinal (GI) complication seen in COVID-19 is GI hemorrhage. In one series, 4% of critically ill COVID-19 patients had GI bleeding [50]. Mesenteric ischemia is another potentially catastrophic complication, the incidence of which in critically ill patients may be as high as 3.8–4% [51]. Other serious complications include acute pancreatitis, acute cholecystitis, Budd–Chiari syndrome, ileus, feed intolerance and acute colonic pseudo-obstruction, although the true incidence is unclear [52,53]. In a retrospective cohort study, 45% of COVID-19 patients had mild liver injury, 21% moderate and 6.4% severe. Severe acute liver injury was associated with high ferritin (OR: 2.40) and IL-6 levels (OR: 1.45) [54]. Clostridium difficile infection is also of concern in hospitalized COVID-19 patients, particularly those on broad-spectrum antibiotics. It should be suspected in all hospitalized COVID-19 patients who develop sudden abdominal pain, fever, leukocytosis and diarrhea.

## Neuromuscular & musculoskeletal complications

Critical illness myo-neuropathy in COVID-19 is probably underdiagnosed and must be anticipated in critically ill patients, especially those who required prolonged mechanical ventilation, received corticosteroids and neuromuscular blockade [38]. Such patients are also at risk for prolonged ICU stay and thromboembolic events [55]. More than 23 cases of myositis have been reported, with presentations ranging from muscle weakness to dermatomyositis with the classic rash [56]. Paraspinal myositis can interfere with ambulation and rehabilitation. Rhabdomyolysis is another complication that has to be identified and treated early, especially since it can lead to severe acute kidney injury requiring renal replacement therapy and has a mortality rate close to 50% [56].

## Endocrinological complications

COVID-19 infection can precipitate diabetic ketoacidosis (DKA), hyperosmolar hyperglycemic state (HHS) and severe insulin resistance [57]. This problem is compounded by the use of corticosteroids for the treatment of COVID-19. Therefore, strict glycemic control is essential. These complications must be anticipated in nondiabetics as well. In a systematic review, 23% of COVID-19 patients who developed DKA or HHS did not have pre-existing diabetes mellitus [58]. It must also be noted that DKA is a strong risk factor for the development of mucormycosis. Bilateral adrenal hemorrhage and adrenal insufficiency have also been reported in COVID-19 and may require emergent resuscitative measures [59,60].

## Dermatologic complications

A wide range of dermatologic complications have been described in COVID-19. In moderate-to-severe disease, vesicular, urticarial, morbilliform eruptions and chilblain-like or perniosis-like lesions may be seen [61]. Critically ill patients may have a livedoid rash or retiform purpura [61]. Erythema multiforme/Stevens–Johnson syndrome can also be seen and may be life threatening [62]. In addition, 60–74% of children with the multisystem inflammatory syndrome (MIS-C) have mucocutaneous involvement consisting of conjunctival injection, palmoplantar erythema, lip hyperemia, periorbital erythema, strawberry tongue and malar erythema.

## Rheumatologic & other rare complications

More than 60 cases of hemophagocytic lymphohistiocytosis (HLH) associated with COVID-19 have been published to date [63]. It is postulated that HLH, multisystem inflammatory syndrome in children (MIS-C) and the hyper-inflammatory syndrome (‘cytokine storm’) seen in adults share similar, if not common pathogenetic pathways. There have also been case reports of COVID-19 patients developing vasculitis of small, medium and large vessels [64]. These can present in many ways and must be borne in mind while evaluating COVID-19 patients with atypical presentations. Reactive arthritis and new onset-inflammatory arthritis have also been described after COVID-19 [65,66]. Other rare complications of COVID-19 reported in the literature include Evans syndrome, pseudo-appendicitis, cold-agglutinin autoimmune hemolytic anemia and Kikuchi–Fujimoto disease [67–70].

## Conclusion

Most countries are experiencing multiple waves of COVID-19. While the mortality associated with this disease is profound, it is also essential to consider the complications of COVID-19 that do not result in death. These have a significant impact on a survivor's quality of life and are likely to have a significant economic impact. In conclusion, COVID-19 is a multi-system disease in the truest sense with a myriad of complications. It is imperative that physicians are aware of these complications and are equipped to respond to the same. Such holistic care requires a multi-disciplinary team.

## Future perspective

We must also take this opportunity to look back and learn from our missteps. A systematic investment of resources and compliance with good clinical practices into measures to prevent a global pandemic in the future is a need of the hour. The present pandemic has also made clear the divide between the scientific community and the lay person in terms of access to scientific literature. More efforts have to be made to simplify the enormous amounts of data that are being generated into easy-to-understand and meaningful messages for the public.

While this paper has focused on in-hospital complications, there are a multitude of short and long-term complications of this disease and the data continues to evolve on that front. Given the course of the pandemic and its perpetuation in waves, hitherto unidentified complications may be discovered in the future. It is also possible that some rare complications related to COVID-19 vaccines may surface, given the sheer scale of vaccination. This will naturally lead to trials aimed at identifying the appropriate management of such complications. It is entirely possible that the study of COVID-19, its complications and their management may evolve into a specialized branch of medicine and a major public health area of interest.
